# Genomic regions associated with host response to porcine reproductive and respiratory syndrome vaccination and co-infection in nursery pigs

**DOI:** 10.1186/s12864-017-4182-8

**Published:** 2017-11-13

**Authors:** Jenelle R. Dunkelberger, Nick V. L. Serão, Ziqing Weng, Emily H. Waide, Megan C. Niederwerder, Maureen A. Kerrigan, Joan K. Lunney, Raymond R. R. Rowland, Jack C. M. Dekkers

**Affiliations:** 1 Department of Animal Science, Iowa State University, Ames, IA 50011 USA; 2 Topigs Norsvin USA, Burnsville, MN 55337 USA; 3 ABS Global Inc., DeForest, WI 53532 USA; 4 The Seeing Eye Inc., Morristown, NJ 07960 USA; 5 Department of Diagnostic Medicine/Pathobiology, College of Veterinary Medicine, Kansas State University, Manhattan, KS 66506 USA; 6 USDA, ARS, BARC, APDL, Beltsville, MD 20705 USA

**Keywords:** Functional annotation, Genome-wide association study, PCV2, PRRS, Quantitative trait locus, Swine, WUR10000125

## Abstract

**Background:**

The WUR1000125 (**WUR**) single nucleotide polymorphism (**SNP**) can be used as a genetic marker for host response to porcine reproductive and respiratory syndrome (**PRRS**), PRRS vaccination, and co-infection with porcine circovirus type 2b (**PCV2b**). Objectives of this study were to identify genomic regions other than WUR associated with host response to PRRS vaccination and PRRSV/PCV2b co-infection and regions with a different effect on host response to co-infection, depending on previous vaccination for PRRS.

**Methods:**

Commercial crossbred nursery pigs were pre-selected for WUR genotype (*n* = 171 AA and 198 AB pigs) where B is the dominant and favorable allele. Half of the pigs were vaccinated for PRRS and 4 weeks later, all pigs were co-infected with PRRS virus and PCV2b. Average daily gain (**ADG**) and viral load (**VL**) were quantified post vaccination (**Post Vx**) and post co-infection (**Post Co-X**). Single-SNP genome-wide association analyses were then conducted to identify genomic regions associated with response to vaccination and co-infection.

**Results:**

Multiple SNPs near the major histocompatibility complex were significantly associated with PCV2b VL (*−log*_*10*_ *P ≥* 5.5), regardless of prior vaccination for PRRS. Several SNPs were also significantly associated with ADG Post Vx and Post Co-X. SNPs with a different effect on ADG, depending on prior vaccination for PRRS, were identified Post Vx (*−log*_*10*_ *P =* 5.6) and Post Co-X (*−log*_*10*_ *P =* 5.5). No SNPs were significantly associated with vaccination VL (−log_10_ *P ≤* 4.7) or PRRS VL (−log_10_ *P ≤* 4.3). Genes near SNPs associated with vaccination VL, PRRS VL, and PCV2b VL were enriched (*P ≤* 0.01) for immune-related pathways and genes near SNPs associated with ADG were enriched for metabolism pathways (*P ≤* 0.04). SNPs associated with vaccination VL, PRRS VL, and PCV2b VL showed overrepresentation of health QTL identified in previous studies and SNPs associated with ADG Post Vx of Non-Vx pigs showed overrepresentation of growth QTL.

**Conclusions:**

Multiple genomic regions were associated with PCV2b VL and ADG Post Vx and Post Co-X. Different SNPs were associated with ADG, depending on previous vaccination for PRRS. Results of functional annotation analyses and novel approaches of using previously-reported QTL support the identified regions.

**Electronic supplementary material:**

The online version of this article (10.1186/s12864-017-4182-8) contains supplementary material, which is available to authorized users.

## Background

Guanylate binding protein 5 (***GBP5***), located on *Sus scrofa* chromosome (**SSC**) 4, was identified as a major gene for host response to porcine reproductive and respiratory syndrome (**PRRS**) [1, 2]. Since the causative mutation for *GBP5* does not appear on commercial genotyping platforms, the single nucleotide polymorphism (**SNP**) WUR10000125 (**WUR**), which is in complete linkage disequilibrium with the putative causative mutation [2–4] can be used as a genetic marker for this mutation.

Since the identification of this quantitative trait locus (**QTL**), the effect of WUR has been associated with host response to PRRS virus (**PRRSV**)-infection following infection with two different PRRSV isolates [3–6], PRRS vaccination [7], and co-infection with PRRSV and porcine circovirus type 2b (**PCV2b**) [7]. In addition, WUR was associated with PCV2b viral load (**VL**) following PRRSV/PCV2b co-infection for pigs previously vaccinated for PRRS, but not for non-vaccinated pigs [7]. Other genomic regions, including regions on SSC7 and SSC12 have been associated with PCV2b VL following experimental infection with PCV2b only [8].

The first objective of this study was to identify genomic regions other than WUR that are associated with host response to PRRS vaccination and co-infection with PRRSV and PCV2b. A second objective was to identify regions with a different effect on host response to PRRSV/PCV2b-infection, depending on whether pigs were previously vaccinated for PRRS. The final objective was to assess the biological relevance of genomic regions associated with each trait to provide support for, and assign biological function to, these statistically-associated regions.

## Methods

Experiments involving animals and virus were performed in accordance with the Federation of Animal Science Societies Guide for the Care and Use of Agricultural Animals in Research and Teaching, the USDA Animal Welfare Act and Animal Welfare Regulations, or according to the National Institutes of Health’s Guide for the Care and Use of Laboratory Animals, and were approved by the Kansas State University and Iowa State University institutional animal care and use committees and institutional biosafety committees. Animals were humanely euthanized by pentobarbital overdose following the American Veterinary Medical Association (AVMA) guidelines for the euthanasia of animals, and all efforts were made to minimize suffering.

### Animals

A detailed description of the animals used for this study is in Dunkelberger et al. [7]. Briefly, commercial Large White x Landrace crossbred barrows from two experimental co-infection trials (trial 1 *n* = 199; trial 2 *n* = 197) were used. Pigs originated from the same, high health multiplier farm and were pre-selected based on WUR marker genotype: approximately half for the AA genotype (*n* = 184) and the other half for the AB genotype (*n* = 212), where the B allele is the favorable and dominant allele [1]. Pigs were shipped to Kansas State University at weaning (between 18 and 28 days of age) and sorted into one of 2 rooms upon arrival. Within each room, pens were balanced according to WUR genotype and pigs were given 3-4 days to acclimate to their new surroundings before all pigs within one room were vaccinated with a 2-mL dose of a commercial PRRS MLV vaccine (Ingelvac PRRS®, Boehringer Ingelheim Vetmedica Inc., St. Joseph, MO). Four weeks later, all pigs were co-infected with field strains of PRRSV and PCV2b [9]. Pigs were followed for the next 42 days, after which all surviving pigs were humanely euthanized using an intravenous injection of pentobarbital sodium and tissue was collected for genotyping. Body weights were recorded weekly throughout the vaccination (−28 to 0 days post-infection [**dpi**]) and co-infection (0 to 42 dpi) periods on both vaccinated (**Vx**) and non-vaccinated (**Non-Vx**) pigs. Serum samples were collected on Vx pigs following vaccination (**Post Vx**) at −28, −24, −21, −17, −14, and −7 dpi and on both Vx and Non-Vx pigs following co-infection (**Post Co-X**) at 0, 4, 7, 11, 14, 21, 28, 35, and 42 dpi. Serum samples were used to quantify PRRSV and PCV2b viremia using real-time polymerase chain reaction according to Niederwerder et al. [9].

### Traits

A detailed description of the traits analyzed for this study, including descriptive statistics for all traits analyzed, is in Dunkelberger et al. [7]. Briefly, 89 Non-Vx AA, 106 Non-Vx AB, 95 Vx AA, and 106 Vx AB pigs were used for all analyses. ADG was calculated as the regression of body weight on dpi using body weight data from −28 to 0 and 0 to 42 dpi for ADG Post Vx and Post Co-X, respectively. Vaccination VL, PRRS VL, and PCV2b VL were calculated for each individual as the area under the curve of log_10_-transformed viremia from −28 to 0, 0 to 21, and 0 to 42 dpi, respectively.

### Genotype data

Ear tissue was used to genotype pigs from trials 1 and 2 using the GeneSeek-Neogen PorcineSNP80 BeadChip (GeneSeek, Igenity; Lincoln, NE). Quality control of genotype data was performed in the following steps: 1) fixed SNPs were removed, 2) SNP genotypes with a gene call score lower than 0.3 were set to missing, and 3) SNPs missing more than 15% of genotypes were removed. Final genotyping rate, calculated as the percent of SNP genotypes called out of the total number of genotypes, was 99.43%.

For all analyses presented, a genomic relationship matrix (**GRM**) was used to account for relationships among the 369 individuals with both genotypes and phenotypes and was constructed according to VanRaden [10] by centering and scaling genotypes for all individuals across the 61,729 SNPs that remained after quality control.

### Genome-wide association analyses (GWAS)

All GWAS were performed using single-SNP analyses with ASReml 4.0 [11].

#### Univariate GWAS

The following univariate animal model was used to test the effect of SNP genotype on vaccination VL by fitting the effect of one SNP at a time, with animal, litter, and pen (trial) fitted as random effects to account for genetic, common environmental, and random environmental effects, respectively:

**Model [1]**

$$ {\displaystyle \begin{array}{l}{\mathrm{Y}}_{\mathrm{i}\mathrm{jklmn}}=\upmu +{\mathrm{Trial}}_{\mathrm{j}}+{\mathrm{WUR}}_{\mathrm{k}}+{\mathrm{SNP}}_{\mathrm{l}}+\\ {}{\upbeta}_1\ast {\mathrm{WtVx}}_{\mathrm{i}}+{\upbeta}_2\ast {\mathrm{VxAge}}_{\mathrm{i}}+{\upbeta_3}^{\ast}\mathrm{PCV}2\_{0}_{\mathrm{i}}\\ {}+{\mathrm{Animal}}_{\mathrm{i}}+{\mathrm{Litter}}_{\mathrm{m}}+{\mathrm{Pen}}_{\mathrm{n}\left(\mathrm{j}\right)}+{\mathrm{e}}_{\mathrm{i}\mathrm{jklmn}}\end{array}} $$

where Y_ijklm_ = is the observed phenotype; Trial_j_ = fixed effect of the j^th^ trial (1 or 2); WUR_k_ = fixed effect of WUR SNP genotype (AA or AB); SNP = fixed effect of the l^th^ genotype (AA, AB, or BB); β_p_ = partial regression coefficient for the covariate weight at −28 dpi (WtVx) (*P =* 1), age at −28 dpi (VxAge) (*P =* 2), and PCV2b viremia at 0 dpi (*P =* 3); Animal_i_ = random animal genetic effect of the i^th^ individual, with a variance-covariance structure proportional to the GRM with the assumption ~ $\mathrm{N}\Big(0,\mathbf{G}{\upsigma}_{\upalpha}^2$); Litter = random litter effect (123 levels), assumed to be $\sim \mathrm{N}\Big(0,\mathbf{I}{\upsigma}_{\mathrm{l}}^2$); and Pen = random effect of pen nested within trial (40 levels), assumed to be $\sim \mathrm{N}\Big(0,\mathbf{I}{\upsigma}_{\mathrm{p}}^2$). Interaction effects of trial with each fixed effect were fitted but removed since they were not significant (*P >* 0.10).

Including WUR genotype in the model as a fixed effect allowed for the identification of regions other than WUR associated with host response to PRRS vaccination, while simultaneously accounting for the effect of this marker. Level of PCV2b viremia at 0 dpi was fitted as a covariate because 37 (24 Non-Vx and 13 Vx) pigs had non-zero PCV2b viremia titers at 0 dpi, suggesting that they were exposed to PCV2b prior to entering the facility, likely from their mothers; all phenotypes were adjusted to PCV2_0 = 0 to model the situation that all pigs were negative for PCV2b at 0 dpi.

#### Bivariate GWAS

To identify the effect of vaccination on the effect of genomic regions for host response to co-infection, bivariate animal models were used to analyze ADG Post Vx, PRRS VL, PCV2b VL, and ADG Post Co-X of Vx versus Non-Vx groups as two separate traits, as described in Dunkelberger et al. [7]. For each bivariate GWAS, the following model was used to test the effect of each SNP averaged across vaccination status (**main effect**) and the interaction of SNP genotype with vaccination status (**interaction effect**):

**Model** [2]

$$ \left[\begin{array}{c}{\mathbf{y}}_{\mathbf{N}}\\ {}{\mathbf{y}}_{\mathbf{V}}\end{array}\right]=\left[\begin{array}{cc}{\mathbf{X}}_{\mathbf{N}}& 0\\ {}0& {\mathbf{X}}_{\mathbf{V}}\end{array}\right]\left[\begin{array}{c}{\mathbf{b}}_{\mathbf{N}}\\ {}{\mathbf{b}}_{\mathbf{V}}\end{array}\right]+\left[\begin{array}{cc}{{\mathbf{W}}_{\mathbf{i}}}_{\mathbf{N}}& 0\\ {}0& {{\mathbf{W}}_{\mathbf{i}}}_{\mathrm{V}}\end{array}\right]\left[\begin{array}{c}{{\mathbf{g}}_{\mathbf{i}}}_{\mathbf{N}}\\ {}{{\mathbf{g}}_{\mathbf{i}}}_{\mathbf{V}}\end{array}\right]+\left[\begin{array}{cc}{\mathbf{Z}}_{{\boldsymbol{\upalpha}}_{\mathbf{N}}}& 0\\ {}0& {\mathbf{Z}}_{{\boldsymbol{\upalpha}}_{\mathbf{V}}}\end{array}\right]\left[\begin{array}{c}{\boldsymbol{\upalpha}}_{\mathbf{N}}\\ {}{\boldsymbol{\upalpha}}_{\mathbf{V}}\end{array}\right]+\left[\begin{array}{cc}{\mathbf{Z}}_{{\mathbf{l}}_{\mathbf{N}}}& 0\\ {}0& {\mathbf{Z}}_{{\mathbf{l}}_{\mathbf{V}}}\end{array}\right]\left[\begin{array}{c}{\mathbf{l}}_{\mathbf{N}}\\ {}{\mathbf{l}}_{\mathbf{V}}\end{array}\right]+\left[\begin{array}{cc}{\mathbf{Z}}_{{\mathbf{p}}_{\mathbf{N}}}& 0\\ {}0& {\mathbf{Z}}_{{\mathbf{p}}_{\mathbf{V}}}\end{array}\right]\left[\begin{array}{c}{\mathbf{p}}_{\mathbf{N}}\\ {}{\mathbf{p}}_{\mathbf{V}}\end{array}\right]+\left[\begin{array}{c}{\mathbf{e}}_{\mathbf{N}}\\ {}{\mathbf{e}}_{\mathbf{V}}\end{array}\right] $$

where subscripts N and V represent a trait recorded on Non-Vx and Vx pigs, respectively; **y**_N_(**y**_V_) = vector of phenotypes; **X**_N_(**X**_V_) = design matrix of fixed effects (same as for Model [**1**]); **b**_N_(**b**_V_) = vector of solutions for fixed effects; **W**_iN_(**W**_iV_) = matrix of fixed genotype effects for the i^th^ SNP genotype and **g**_iN_(**g**_iV_) = corresponding vector of solutions; ${\mathbf{Z}}_{{\boldsymbol{\upalpha}}_{\mathbf{N}}}$(${\mathbf{Z}}_{{\boldsymbol{\upalpha}}_{\mathbf{V}}}$) = design matrix of random genetic effects and **α**_N_(**α**_V_) = corresponding vector of solutions;${\mathbf{Z}}_{{\mathbf{1}}_{\mathbf{N}}}\left({\mathbf{Z}}_{{\mathbf{1}}_{\mathbf{V}}}\right)$= design matrix of random litter effects and **l**_N_(**l**_V_) = corresponding vector of solutions; ${\mathbf{Z}}_{{\mathbf{p}}_{\mathbf{N}}}\left({\mathbf{Z}}_{{\mathbf{p}}_{\mathbf{V}}}\right)$= design matrix of random pen effects and **p**_N_(**p**_V_) = corresponding vector of solutions. The covariance matrix of random animal genetic, litter, pen, and residual effects was specified as follows:

$$ \mathrm{Var}\left[\begin{array}{c}{\boldsymbol{\alpha}}_N\\ {}{\boldsymbol{\alpha}}_V\\ {}{\boldsymbol{l}}_N\\ {}{\boldsymbol{l}}_{\boldsymbol{V}}\\ {}{\boldsymbol{p}}_N\\ {}{\boldsymbol{p}}_V\\ {}{\boldsymbol{e}}_N\\ {}{\boldsymbol{e}}_V\end{array}\right]=\left[\begin{array}{cccccccc}{\mathbf{G}}_{\boldsymbol{N}}{\sigma}_{\alpha\ N}^2& {\boldsymbol{G}}_{\mathbf{NV}}{\sigma}_{\alpha\ N,V}& 0& 0& 0& 0& 0& 0\\ {}{\boldsymbol{G}}_{\mathbf{NV}}{\sigma}_{\alpha\ N,V}& {\mathbf{G}}_{\boldsymbol{V}}{\sigma}_{\alpha\ V}^2& 0& 0& 0& 0& 0& 0\\ {}0& 0& \mathbf{I}{\sigma}_{l\ N}^2& \mathbf{I}{\sigma}_{l\ N,V}& 0& 0& 0& 0\\ {}0& 0& \mathbf{I}{\sigma}_{l\ N,V}& \mathbf{I}{\sigma}_{l\ V}^2& 0& 0& 0& 0\\ {}0& 0& 0& 0& \mathbf{I}{\sigma}_{p\ N}^2& 0& 0& 0\\ {}0& 0& 0& 0& 0& \mathbf{I}{\sigma}_{p\ V}^2& 0& 0\\ {}0& 0& 0& 0& 0& 0& \mathbf{I}{\sigma}_{e\ N}^2& 0\\ {}0& 0& 0& 0& 0& 0& 0& {\mathbf{I}\sigma}_{e\ V}^2\end{array}\right] $$

where **G** represents the GRM for the Non-Vx(**N**) and Vx(**V**) individuals and **I** represents the identity matrix. For all analyses, covariances were allowed between traits for the animal genetic effect and litter effect. However, covariances were constrained to 0 for pen effects and residuals since Vx and Non-Vx pigs were allocated to different rooms and no pig had records for both traits.

#### Correction for multiple testing

Multiple test correction was performed to determine the appropriate significance threshold to interpret GWAS results. First, principal component analysis was used to determine the number of independent tests (i.e. SNPs) per chromosome as the number of principal components required to capture 99.5% of the variation [6, 12] using the *princomp* function of R software [13]. Results are presented in Additional file 1: Table S1. Since the number of individuals used for analyses serves as an upper bound for the number of principal components that can be identified, chromosomes were divided into segments so that for each segment, the number of SNPs was fewer than the total number of individuals (i.e. *n* < 369). The number of independent tests per chromosome segment was then summed to calculate the total number of independent tests across the genome. Unmapped SNPs were not included in this calculation. Bonferroni correction was then applied using the following equation:

$$ p-\mathrm{value}=\frac{\upalpha}{\mathrm{number}\  \mathrm{of}\  \mathrm{independent}\  \mathrm{tests}\  \mathrm{across}\  \mathrm{the}\  \mathrm{genome}} $$

where α = 0.10 and the number of independent tests for the whole genome = 26, 272. The resulting genome-wise significance –log_10_ *p*-value threshold was 5.4.

### Pathway analyses

Pathway analyses were conducted to identify protein pathways that were enriched for genes near SNPs associated with each trait according to the procedure described by Waide et al. [6]. First, lists of SNPs at three –log_10_ p-value thresholds (2, 2.5, and 3, referred to as SNP lists **T2**, **T2.5**, and **T3**, respectively) for the combined additive and dominance effect (2-df test) of each SNP were generated for each trait. Next, 1-megabase (**Mb**) windows were constructed for each SNP, which spanned 0.5 Mb on either side of each SNP. Then, for each SNP, Ensembl IDs (http://www.ensembl.org) [14] were obtained for all genes contained within or flanking either side of the 1-Mb SNP window. These Ensembl IDs were then used to perform a statistical overrepresentation analysis using the gene ontology (**GO**) Slim feature of the PANTHER software [15]. Compared to using the entire GO term database, GO Slim uses a limited set of GO terms (550 versus 45,237 total annotations) to provide a more general list of protein pathways that map to gene IDs (http://www.pantherdb.org) [16]. A custom background gene list was used, which consisted of all genes that mapped to 0.5 Mb on either side of each of the 61,729 SNPs used for GWAS. *P*-values for statistical overrepresentation of protein pathways were corrected by PANTHER software using the Bonferroni multiple testing correction method. The number of SNPs and genes corresponding to each SNP list for each trait are shown in Additional file 1: Tables S2 and S3.

### QTL test

A second type of analysis, hereafter referred to as the **QTL Test**, was also used to provide evidence for regions associated with each trait in this study. A master list of previously reported QTL for all traits in the pig genome was downloaded from the QTL database for pigs at *animalgenome.org* [17]*.* In the database, each QTL entry is assigned to one of the following trait categories: exterior, health, meat and carcass, production, and reproduction. In total, 16,032 QTL entries were downloaded. Filtering of the master list was then performed according to the following steps: duplicate QTL entries (i.e. entries for the same trait, location, and publication) were removed, QTL for the same trait from the same study with overlapping positions were concatenated, QTL spanning more than 5 Mb were removed, and all ADG, body weight, and growth-related QTL, which belong to the “production” category, were re-assigned to a new category entitled “growth”. After filtering, 9892 unique QTL entries remained.

The filtered master QTL list was then used to construct a list of all QTL that mapped to each SNP. A QTL was mapped to a SNP if the QTL was either entirely contained within or entirely overlapped the 1-Mb SNP window. This list was used to determine the number of health and growth QTL that mapped to SNPs associated with VL and ADG traits, respectively. These lists were then used to assess overrepresentation of health or growth QTL mapping to SNPs for each list.

Using QTL_I_ as the trait QTL (i.e. QTL for health or growth) mapped to one of the three SNP lists for a trait (i.e. T2, T2.5 or T3), QTL_T_ as the list of all QTL (of all QTL types) mapped to one of the three SNP lists, and QTL_G_ as the list of all QTL (for all QTL types) throughout the genome, the null hypothesis (H_0_) for this test was that the ratio P_1_ = QTL_I_:QTL_T_ was equal to the ratio P_2_ = QTL_I_:QTL_G_. The alternative hypothesis (H_a_) was that P_1_ > P_2._

*P*-values for the QTL Test were calculated as the probability of identifying QTL_I_ or more trait QTL for a given SNP list under H_0_ using the binomial distribution, as follows:

$$ \mathrm{p}-\mathrm{value}=\sum \limits_{\mathrm{x}=\mathrm{k}}^{\mathrm{K}}\left(\begin{array}{c}\mathrm{K}\\ {}\mathrm{x}\end{array}\right){\mathrm{P}}^{\mathrm{x}}{\left(1-\mathrm{P}\right)}^{\mathrm{K}-\mathrm{x}} $$

where k = QTL_I_; K = QTL_T_; and P = P_2_. Analyses were performed separately for health and growth QTL for each SNP list.

### SNP test

A third test, hereafter referred to as the **SNP Test**, was also performed to assess overrepresentation of health or growth QTL, but using slightly different information than the QTL Test. Using SNP_I_ as the number of unique SNPs within a health or growth QTL (i.e. QTL_I_), SNP_T_ as the number of SNPs in each SNP list (i.e. T2, T2.5, or T3), and SNP_G_ the number of SNPs across the genome (i.e. 61,729), the H_0_ for this test was that P_3_ = SNP_I_:SNP_T_ was equal to P_4_ = SNP_I_:SNP_G_ (H_0_: P_3_ = P_4_). The alternative hypothesis (H_a_) was that P_3_ > P_4_. P-values for the SNP Test were also calculated using the above binomial distribution but using k = SNP_I_, K = SNP_T_, and P = P_4_.

## Results

### GWAS post vaccination

#### Vaccination viral load

**Fig. 1:**
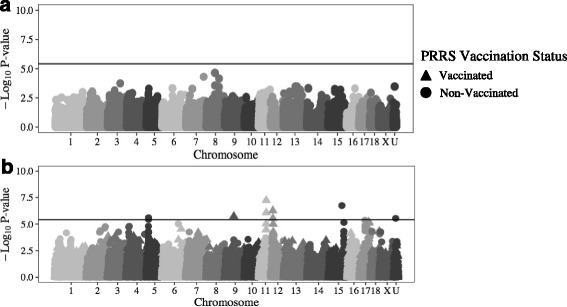
Significance of GWAS results for vaccination VL (**a**) and ADG (**b**) following vaccination

No SNPs were significantly associated with vaccination VL (maximum *-log*_*10*_ *P* = 4.65) (Fig. 1a).

#### Average daily gain

Five total SNPs, located on SSC9 at 52 Mb (i.e. 9_52), 11_53, and from 12_5 to 12_6 (Fig. 1b) were significantly (*−log*_*10*_ *P ≥* 5.6) associated with ADG Post Vx for Vx pigs (Table 1). A different set of SNPs, including SNPs located at 15_129 and 5_5 (Fig. 1b) were significantly (*−log*_*10*_ *P ≥* 5.4) associated with ADG Post Vx of Non-Vx pigs (Table 1).

**Table 1:** SNPs associated with host response to PRRS vaccination and PRRSV/PCV2b co-infection

Infection Period	Trait	VxStatus^a^	SNP Name	Chromosome	Mb^b^	*P*-value^c^
Post Vaccination	Vaccination VL^d^	Vx	–	–	–	–
	ADG^e^	Vx	ALGA0052956	9	52	5.7
			WU_10.2_11_53143619	11	53	7.2
			ALGA0062289	11	53	6.1
			ASGA0083776	12	5	6.3
			ISU10000072	12	6	5.6
		Non-Vx	WU_10.2_5_5635354	5	5	5.6
			WU_10.2_5_5693454	5	5	5.4
			MARC0034977	15	129	6.8
Post Co-Infection	PRRS VL	Vx	–	–	–	–
		Non-Vx	–	–	–	–
	PCV2b VL	Vx	–	–	–	–
		Non-Vx	H3GA0020199	7	20	6.1
	ADG	Vx	MARC0021766	1	47	6.9
			ALGA0077929	14	61	5.5
			H3GA0040428	14	62	5.5
		Non-Vx	H3GA0020408	7	27	5.8
			WU_10.2_15_140171163	15	140	5.4

^a^VxStatus, vaccination status: Pigs were either vaccinated (**Vx**) or not (**Non-Vx**) for PRRS prior to co-infection of PRRSV with PCV2b 28 days later

^b^Mb, megabase

^c^ *P*-value: -log_10_ *p*-value

^d^VL: calculated as the area under the curve from −28 to 0, 0 to 21, or 0 to 42 dpi for vaccination VL, PRRS VL, and PCV2b VL, respectively

^e^ADG: calculated as the regression of body weight on dpi post vaccination and post PRRSV/PCV2b co-infection

**Fig. 2:**
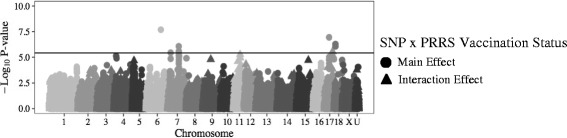
Significance of GWAS results for main/interaction effects for ADG following vaccination

Seven SNPs had a significant (*−log*_*10*_ *P ≥* 5.6) main effect on ADG Post Vx (Table 2). These SNPs were located at 6_108, 7_81 to 7_82, 17_32, and 18_4 (Fig. 2). One of the only two SNPs with a significant interaction effect identified in this study was also associated with ADG Post Vx (*−log*_*10*_ *P* = 5.6) (Fig. 2), and was located at 17_57 (Table 2). However, the effect of this SNP on ADG Post Vx was not significant when analyzed for Vx (*−log*_*10*_ *P* = 5.1) or Non-Vx (*−log*_*10*_ *P* = 1.0) pigs separately (Fig. 1b).

**Table 2:** SNPs with significant main/interaction effects with vaccination status following PRRS vaccination and PRRSV/PCV2b co-infection

Infection Period	Trait	Effect^a^	SNP Name	Chromosome	Mb^b^	*P*-value^c^
Post Vaccination	ADG^d^	Main	ALGA0036437	6	108	7.7
			ALGA0107326	7	81	6.1
			MARC0056209	7	81	5.6
			ALGA0042683	7	82	5.6
			WU_10.2_17_32849954	17	32	6.9
			WU_10.2_18_4027654	18	4	6.3
			WU_10.2_18_4233046	18	4	6.1
		Interaction	ASGA0077518	17	57	5.6
Post Co-Infection	PRRS VL^e^	Main	–	–	–	–
		Interaction	–	–	–	–
	PCV2b VL	Main	DRGA0007276	7	19	6.2
			H3GA0020199	7	20	8.7
			ASGA0032282	7	32	5.5
			MARC0060135	7	41	7.3
		Interaction	–	–	–	–
	ADG	Main	MARC0021766	1	47	7.1
			MARC0059955	7	24	7.7
			WU_10.2_15_140171163	15	140	6.0
		Interaction	WU_10.2_4_6084304	4	6	5.5

^a^Effect: The effect of SNP across groups vaccinated, or not, for PRRS (**main**) versus the effect of SNP interacting with vaccination status (**interaction**)

^b^Mb, megabase

^c^ *P*-value: -log_10_ p-value

^d^ADG: calculated as the regression of body weight on dpi post PRRS vaccination and post PRRSV/PCV2b co-infection

^e^VL: calculated as the area under the curve from −28 to 0, 0 to 21, or 0 to 42 dpi for vaccination VL, PRRS VL, and PCV2b VL, respectively

### GWAS post co-infection

#### Viral load

**Fig. 3:**
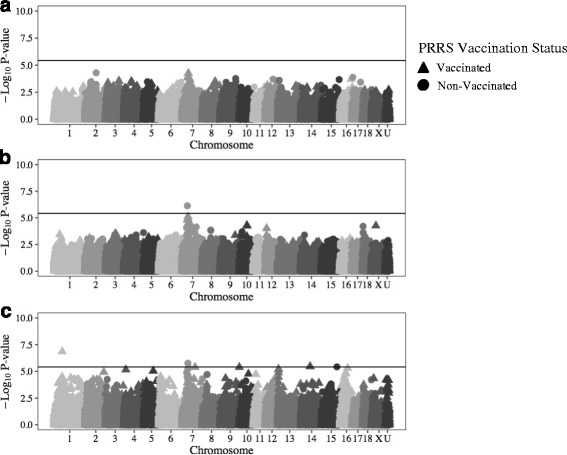
GWAS results for PRRS VL (**a**), PCV2b VL (**b**), and ADG (**c**) following co-infection

Genomic regions associated with host response to co-infection are presented in Fig. 3. One SNP, located at 7_20 (Fig. 3b) was significantly (*−log*_*10*_ *P =* 6.1) associated with PCV2b VL of Non-Vx pigs (Table 1). No SNPs were significantly associated with PCV2b VL of Vx pigs (*−log*_*10*_ *P ≤* 5.1)(Fig. 3b), PRRS VL of Vx pigs (*−log*_*10*_ *P ≤* 4.3)(Fig. 3a), or PRRS VL of Non-Vx pigs (*−log*_*10*_ *P ≤* 4.3) (Fig. 3a).

**Fig. 4:**
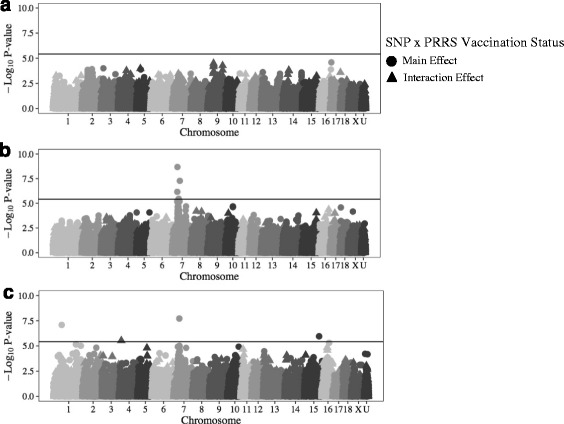
Main/interaction effects for PRRS VL (**a**), PCV2b VL (**b**), and ADG (**c**) following co-infection

SNPs with a significant main effect on host response to co-infection were identified for some traits Post Co-X (Fig. 4). SNPs associated with main and interaction effects of SNP for PRRS VL, PCV2b VL, and ADG are presented in Fig. 4a, b, and c, respectively. Four SNPs had significant (*−log*_*10*_ *P ≥* 5.5) main effects on PCV2b VL (Table 2), all of which were located on SSC7 at 19, 20, 32, and 41 Mb (Fig. 4b). Of these SNPs, the effect of SNP H3GA0020199 (7_20) was significant for PCV2b VL of Non-Vx pigs (*−log*_*10*_ *P* = 6.1), but not Vx pigs (*−log*_*10*_ *P* = 4.8) (Fig. 3b). The interaction effect of SNP genotype by VxStatus was not significant for any SNP for analysis of PCV2b VL (*−log*_*10*_ *P ≤* 4.4) (Fig. 3b). No SNPs had a significant (*−log*_*10*_ *P ≤* 4.6) main or interaction effect on PRRS VL Post Co-X (Fig. 4a).

#### Average daily gain

Three SNPs, located at 1_47 and 14_61 to 14_62 (Fig. 3c) were significantly (*−log*_*10*_ *P ≥* 5.5) associated with ADG Post Co-X of Vx pigs (Table 1). SNPs at 7_27 and 15_140 (Fig. 3c) were significantly (*−log*_*10*_ *P ≥* 5.4) associated with ADG Post Co-X of Non-Vx pigs (Table 1).

Three SNPs had a significant (−*log*_*10*_ *P ≥* 6.0) main effect on ADG Post Co-X (Table 2). These SNPs were located at 1_47, 7_24, and 15_140 (Fig. 4c). Of these SNPs, MARC0021766 (1_47) was also significant for ADG Post Co-X of Vx pigs (*−log*_*10*_ *P* = 6.9), but not Non-Vx pigs (*−log*_*10*_ *P* = 0.5) (Fig. 3c). Conversely, WU_10.2_15_140171163 (15_140) was significantly (*−log*_*10*_ *P* = 5.4) associated with ADG Post Co-X for Non-Vx pigs, but not Vx pigs (*−log*_*10*_ *P* = 1.0) (Fig. 3c).

The second SNP (WU_10.2_4_6084304) with a significant (*−log*_*10*_ *P* = 5.5) interaction effect of SNP genotype by VxStatus identified in this study was associated with ADG Post Co-X (Table 2) located at 4_6 (Fig. 4c). However, the effect of this SNP was not significant for ADG Post Co-X when analyzed separately for Vx (*−log*_*10*_ *P* = 5.2) or Non-Vx (*−log*_*10*_ *P* = 0.5) pigs (Fig. 3c).

### Pathway analyses post vaccination

For each trait, only GO terms that were significantly (*P <* 0.05 following Bonferroni correction) associated with genes near SNPs for each SNP list (i.e. T2, T2.5, and T3) are presented. These significant associations include several protein pathways that were significantly underrepresented, which are presented but will not be discussed. The number of mapped Ensembl IDs and the total number of Ensembl IDs (i.e. mapped and unmapped) are presented in Additional file 1: Table S2 for each trait analyzed separately by VxStatus and in Additional file 1: Table S3 for the main and interaction effects of SNP genotype by VxStatus.

#### Vaccination viral load

GO terms enriched for genes near SNPs for host response to PRRS vaccination are presented in Table 3. Genes near SNPs associated with vaccination VL were enriched for pathways related to cell proliferation, response to stimuli/stress, behavior, cell movement, and cell signaling.

**Table 3:** Significantly enriched GO terms for genes near SNPs associated with traits following PRRS vaccination

Trait	SNP List^a^	GO term	Fold change	*P*-value^b^
Vaccination VL	2	Sensory perception of chemical stimulus	0.53	4.9E-2
	2.5	Behavior	7.38	4.0E-3
		Response to biotic stimulus	5.98	2.2E-3
		Cell proliferation	3.55	2.4E-2
	3	Behavior	23.09	7.1E-7
		Response to biotic stimulus	18.72	5.4E-8
		Cell proliferation	11.12	5.7E-8
		Locomotion	9.30	1.6E-4
		Cytokine-mediated signaling pathway	7.07	4.4E-4
		Cell surface receptor signaling pathway	2.29	3.5E-2
		Signal transduction	1.96	2.2E-2
		Response to external stimulus	5.82	2.4E-3
		Cellular component movement	4.87	7.4E-4
		Response to stress	3.40	4.8E-3
ADG Non-Vx^c^	2	Sensory perception of smell	0.53	1.7E-2
	2.5	–	–	–
	3	–	–	–
ADG Vx	2	G-protein coupled receptor signaling pathway	0.61	2.4E-2
		Cell surface receptor signaling pathway	0.68	7.4E-3
		Sensory perception of smell	< 0.20	1.2E-17
		Sensory perception of chemical stimulus	0.40	2.4E-8
		Sensory perception	0.55	2.6E-5
		Neurological system process	0.69	3.0E-3
		System process	0.73	2.1E-2
	2.5	Sensory perception of smell	< 0.20	7.5E-9
		Sensory perception of chemical stimulus	0.45	2.4E-2
	3	Sensory perception of smell	< 0.20	4.4E-3

^a^SNP List: Lists of SNPs from the GWAS with a –log_10_ p-value above 2, 2.5 or 3

^b^ *P*-value: Bonferroni corrected *p*-value

^c^Non-Vx, Non-Vaccinated: Pigs were either vaccinated (Vx) or not (Non-Vx) for PRRS

#### Average daily gain

No pathways were significantly enriched for genes near SNPs associated with ADG of Vx or Non-Vx pigs **(**Table 3 **)** or for genes near SNPs associated with ADG Post Vx for the main or interaction effects of SNP (Additional file 1: Table S4).

### Pathway analyses post co-infection

#### Viral load

GO terms enriched for genes near SNPs associated with host response to PRRSV and PCV2b co-infection by VxStatus are presented in Table 4. Immune-related pathways, including the G-protein coupled receptor signaling pathway and chromatin organization pathway were enriched for PRRS VL of Vx pigs. None of these same pathways, but rather, sensory-related pathways, were enriched for PRRS VL of Non-Vx pigs. Chromatin organization was enriched for genes near SNPs for PCV2b VL of Vx and Non-Vx pigs. Additional enriched pathways for Vx pigs included cellular defense response, cell-cell adhesion, and nucleobase-containing compound metabolic process.

**Table 4:** Significantly enriched GO terms for genes near SNPs associated with traits following PRRSV/PCV2b co-infection

Trait	SNP List^a^	GO term	Fold change	P-value^b^
PRRS VL Non-Vx^c^	2	Sensory perception of smell	1.67	3.5E-3
	2.5	Sensory perception	1.85	4.3E-2
	3	–	–	–
PRRS VL Vx	2	Cellular protein modification process	0.53	1.2E-2
		Immune response	0.47	4.9E-2
	2.5	Chromatin organization	2.80	2.7E-2
		G-protein coupled receptor signaling pathway	1.82	2.7E-2
	3	–	–	–
PCV2b VL Non-Vx	2	Sensory perception of chemical stimulus	0.50	2.9E-4
		Sensory perception	0.63	2.5E-2
	2.5	Chromatin organization	2.82	2.4E-2
	3	–	–	–
PCV2b VL Vx	2	Cellular defense response	2.61	1.0E-2
		Cell-cell adhesion	2.26	4.7E-3
		Sensory perception of chemical stimulus	0.54	1.4E-2
		Sensory perception	0.61	4.3E-2
	2.5	Cellular defense response	4.38	1.4E-3
		Chromatin organization	3.75	3.2E-4
		Nucleobase-containing compound metabolic process	1.47	3.6E-2
	3	Cellular defense response	8.52	3.3E-4
ADG Non-Vx	2	Chromatin organization	2.07	3.9E-2
	2.5	Chromatin organization	3.14	1.4E-3
	3	Chromatin organization	4.75	8.5E-6
		Nucleobase-containing compound metabolic process	1.53	3.6E-2
		Primary metabolic process	1.35	3.7E-2
ADG Vx	2	Chromatin organization	1.86	2.5E-2
		JAK-STAT cascade	< 0.20	4.1E-2
	2.5	Cholesterol metabolic process	3.54	4.9E-2
	3	Chromatin organization	2.73	1.1E-2
		Sensory perception of chemical stimulus	0.31	9.8E-4

^a^SNP List: Lists of SNPs from the GWAS with a –log_10_ p-value above 2, 2.5 or 3

^b^ *P*-value: Bonferroni corrected *p*-value

^c^Non-Vx, Non-Vaccinated: Pigs were either vaccinated (Vx) or not (Non-Vx) for PRRS

Genes near SNPs associated with the interaction effect of SNP genotype by VxStatus were enriched for the response to stimulus and chromatin organization pathways for PRRS VL (Additional file 1: Table S5). No pathways were significantly enriched for SNPs associated with the main effect of SNP for PRRS VL. The chromatin organization, response to interferon (**IFN**)-γ, and primary metabolic process pathways were significantly enriched for genes near SNPs associated with the main effect of SNP for PCV2b VL (Additional file 1: Table S6). Only the transport pathway was enriched for the interaction effect of SNP genotype by VxStatus for PCV2b VL (Additional file 1: Table S6).

#### Average daily gain

Consistent with results for PRRS VL of Vx pigs and PCV2b VL of both Vx and Non-Vx pigs, genes near SNPs were significantly enriched for chromatin organization for ADG of Vx and Non-Vx pigs Post Co-X (Table 4). Another significantly enriched pathway for ADG Post Co-X of Vx pigs included the cholesterol metabolic process pathway (Table 4).

Only the chromatin organization pathway was significantly enriched for genes near SNPs associated with the main effect of SNP for ADG Post Co-X (Additional file 1: Table S7)**.** This same pathway was enriched for the interaction effect of SNP genotype by VxStatus, in addition to lipid transport and localization (Additional file 1: Table S7).

### QTL test

The QTL Test was performed to assess overrepresentation of health or growth QTL for SNPs associated with VL or ADG, respectively. Results for analyses of each trait by VxStatus are presented in Table 5 and Additional file 1: Table S8 and S9**.** Results for analyses of the main and interaction effects of SNP for each trait are presented in Tables 6 and 7.

**Table 5:** QTL Test results for SNPs associated with vaccination VL

Trait	SNP List^a^	# SNPs above threshold	# health QTL in region^b^	Total # QTL in region^c^	*P*-value
Vaccination VL	2	392	290	1092	6.5E-14
	2.5	124	37	367	0.99
	3	51	16	100	0.69

^a^SNP List: Lists of SNPs from the GWAS with a –log_10_ *p*-value above 2, 2.5 or 3

^b^The total number of health QTL in the genome (after filtering) is 1732

^c^The total number of QTL in the genome (i.e. across all QTL types and after filtering) is 9892

**Table 6:** QTL Test results for the main/interaction effects of SNPs associated with PRRS and PCV2b VL

Trait	Effect^a^	SNP List^b^	# of SNPs above threshold	# of health QTL in region^c^	Total # of QTL in region^d^	*P*-value
PRRS VL	Main	2	528	189	1046	0.33
		2.5	168	64	382	0.67
		3	37	34	83	4.6E-7
	Interaction	2	516	246	1239	0.02
		2.5	159	79	442	0.44
		3	42	2	97	0.99
PCV2b VL	Main	2	823	372	1943	0.03
		2.5	350	260	1076	2.3E-8
		3	160	162	609	1.5E-8
	Interaction	2	520	182	1140	0.92
		2.5	156	42	399	0.99
		3	44	9	126	0.99

^a^Effect: The effect of SNP across groups vaccinated, or not, for PRRS (main) or the effect of SNP interacting with PRRS vaccination status (interaction)

^b^SNP List: Lists of SNPs from the GWAS with a –log_10_ p-value above 2, 2.5 or 3

^c^The total number of health QTL in the genome (after filtering) is 1732

^d^The total number of QTL in the genome (i.e. across all QTL types and after filtering) is 9892

**Table 7:** QTL Test results for the main/interaction effects of SNPs associated with ADG

Infection Period	Effect^a^	SNP List^b^	# of SNPs above threshold	# of growth QTL in region^c^	Total # QTL in region^d^	*P*-value
Post Vaccination	Main	2	902	85	1633	0.32
		2.5	365	45	643	0.01
		3	141	23	300	0.03
	Interaction	2	687	62	1323	0.68
		2.5	251	20	443	0.69
		3	110	11	230	0.58
Post Co-Infection	Main	2	870	107	2069	0.32
		2.5	364	55	1065	0.38
		3	176	23	502	0.67
	Interaction	2	682	77	1519	0.42
		2.5	291	29	536	0.33
		3	120	20	305	0.12

^a^Effect: The effect of SNP across groups vaccinated, or not, for PRRS (main) or the effect of SNP interacting with PRRS vaccination status (interaction)

^b^SNP List: Lists of SNPs from the GWAS with a –log_10_ p-value above 2, 2.5 or 3

^c^The total number of growth QTL in the genome (after filtering) is 488

^d^The total number of QTL in the genome (i.e. across all QTL types after filtering) is 9892

#### Viral load

Results indicated that SNPs associated with vaccination VL were significantly (*P* = 6.5E-14) overrepresented for health QTL for T2 (Table 5). SNPs associated with the main effect of SNP genotype on PRRS VL were significantly (*P* = 4.6E-7) overrepresented for health QTL for T3 (Table 6). The latter result was driven by significant (*P* = 7.9E-7) overrepresentation of health QTL for SNPs associated with PRRS VL of Vx pigs for T3 (Additional file 1: Table S8). SNPs associated with the interaction effect of SNP genotype by VxStatus on PRRS VL were also significantly (*P* = 0.02) overrepresented for health QTL for T2 (Table 6). This result was driven by significant overrepresentation of health QTL for SNPs associated with PRRS VL of both Vx (*P* = 6.7E-12) and Non-Vx (*P* = 3.3E-18) pigs for T2 (Additional file 1: Table S8).

All three lists of SNPs associated with the main effect of SNP on PCV2b VL showed significant (*P* = 0.03, 2.3E-8, and 1.5E-8) overrepresentation of health QTL (Table 6), driven by significant overrepresentation of health QTL for all three lists of SNPs for PCV2b VL for Vx (*P* = 6.7E-5 to 6.0E-4) and Non-Vx (*P* = 1.7E-9 to 4.0E-4) pigs (Additional file 1: Table S8).

#### Average daily gain post vaccination

Results indicated little evidence of overrepresentation of growth QTL for SNPs associated with ADG Post Vx of Vx (*P ≥* 0.15) or Non-Vx (*P ≥* 0.04) pigs (Additional file 1: Table S9). However, SNPs associated with the main effect of SNP on ADG Post Vx were significantly overrepresented for growth QTL for T2.5 (*P* = 0.01) and T3 (*P* = 0.03) (Table 7).

#### Average daily gain post co-infection

SNPs associated with ADG Post Co-X were not significantly overrepresented for growth QTL for Vx (*P ≥* 0.36) or Non-Vx (*P ≥* 0.74) pigs (Additional file 1: Table S9), or for the main (*P ≥* 0.32) or interaction (*P ≥* 0.12) effects of SNP by VxStatus (Table 7).

### SNP test

The SNP Test was conducted to assess overrepresentation of health or growth QTL based on the number of unique SNPs mapping to trait QTL. Results for analysis of each trait by VxStatus are presented in Table 8 and Additional file 1: Table S10 and S11 and results for the main and interaction effects of SNP by VxStatus are presented in Tables 9 and 10.

**Table 8:** SNP Test results for SNPs associated with PRRS vaccination VL

Trait	SNP List^a^	# SNPs mapping to health QTL in SNP list^b^	# SNPs in SNP list^c^	*P*-value
Vaccination VL	2	125	392	2.2E-5
	2.5	36	124	0.06
	3	14	51	0.26

^a^SNP List: Lists of SNPs from the GWAS with a –log_10_ p-value above 2, 2.5 or 3

^b^The number of unique SNPs mapping to health QTL in the genome is 14,063

^c^The total number of SNPs used for the GWAS was 61,729

**Table 9:** SNP Test results for the main/interaction effects of SNPs associated with PRRS and PCV2b VL

Trait	Effect^a^	SNP List^b^	# SNPs mapping to health QTL in SNP list^c^	# SNPs in SNP list^d^	*P*-value
PRRS VL	Main	2	120	528	0.53
		2.5	27	168	0.99
		3	6	37	0.88
	Interaction	2	155	516	8.4E-5
		2.5	45	159	0.06
		3	3	42	0.99
PCV2b VL	Main	2	221	823	3.0E-3
		2.5	105	350	1.0E-3
		3	52	160	3.0E-3
	Interaction	2	110	520	0.83
		2.5	33	156	0.72
		3	8	44	0.82

^a^Effect: The effect of SNP across groups vaccinated, or not, for PRRS (main) or the effect of SNP interacting with PRRS vaccination status (interaction)

^b^SNP List: Lists of SNPs from the GWAS with a –log_10_ p-value above 2, 2.5 or 3

^c^The number of unique SNPs mapping to health QTL in the genome is 14,063

^d^The total number of SNPs used for the GWAS was 61,729

**Table 10:** SNP Test results for the main/interaction effects of SNPs associated with ADG

Infection Period	Effect^a^	SNP List^b^	# SNPs mapping to growth QTL in SNP list^c^	# SNPs in SNP list^d^	*P*-value
Post Vaccination	Main	2	161	902	0.01
		2.5	87	365	7.1E-6
		3	26	141	0.16
	Interaction	2	103	687	0.53
		2.5	48	251	0.05
		3	36	110	2.8E-6
Post Co-Infection	Main	2	126	870	0.70
		2.5	62	364	0.16
		3	33	176	0.11
	Interaction	2	87	682	0.96
		2.5	33	291	0.97
		3	19	120	0.44

^a^Effect: The effect of SNP across groups vaccinated, or not, for PRRS (main) or the effect of SNP interacting with PRRS vaccination status (interaction)

^b^SNP List: Lists of SNPs from the GWAS with a –log_10_ p-value above 2, 2.5 or 3

^c^The number of unique SNPs mapping to growth QTL in the genome is 9294

^d^The total number of SNPs used for the GWAS was 61,729

#### Viral load

Consistent with results for the QTL Test, SNPs associated with vaccination VL were significantly (*P* = 2.2E-5) overrepresented for health QTL for T2 (Table 8). A tendency (*P* = 0.06) for significant overrepresentation of health QTL was also detected for T2.5, but not T3 (*P* = 0.26) (Table 8). For analysis of the main and interaction effects of SNP genotype by VxStatus on PRRS and PCV2b VL, the same SNP lists that showed significant overrepresentation of health QTL for the QTL Test also showed significant overrepresentation of health QTL for the SNP Test (Table 9). An exception was for SNPs associated with the main effect of SNP genotype on PRRS VL for T3, which was not significant (*P =* 0.88) for the SNP Test (Table 9). SNPs associated with PRRS VL of Vx pigs, PCV2b VL of Non-Vx pigs, and PCV2b VL of Vx pigs were significantly (*P* = 7.0E-12 to 7.0E-3; *P* = 1.1E-4 to 0.02; *P* = 0.04 to 0.01) overrepresented for health QTL (Additional file 1: Table S10). One exception was T3 for PCV2b VL of Vx pigs, which did not show significant (*P* = 0.26) overrepresentation of health QTL (Additional file 1: Table S10).

#### Average daily gain post vaccination

SNPs associated with the main effect of SNP on ADG Post Vx showed significant overrepresentation of growth QTL for T2 (*P* = 0.01) and T2.5 (*P* = 7.1E-6)(Table 10). SNPs associated with the interaction effect of SNP genotype by VxStatus on ADG Post Vx also showed significant overrepresentation of growth QTL for T2.5 (*P* = 0.05) and T3 (*P* = 2.8E-6) (Table 10). For T2, overrepresentation of growth QTL for SNPs associated with the main effect of SNP was driven by significant overrepresentation of growth QTL for Non-Vx pigs for T2 (*P* = 2.0E-4)(Additional file 1: Table S11). Overrepresentation of growth QTL for SNPs associated with the interaction effect of SNP was driven by significant overrepresentation of growth QTL for Non-Vx pigs for T3 (*P* = 8.7E-6) (Additional file 1: Table S11).

#### Average daily gain post co-infection

SNPs associated with the main effect (*P ≥* 0.11) and interaction effect (*P ≥* 0.44) of SNP on ADG Post Co-X were not significantly overrepresented for any of the SNP lists (Table 10). Similarly, growth QTL were not significantly overrepresented for SNPs associated with ADG of Vx (*P ≥* 0.29) or Non-Vx (*P ≥* 0.53) pigs Post Co-X (Additional file 1: Table S11).

## Discussion

This is the first study to identify genomic regions (other than WUR) associated with host response to PRRS MLV vaccination and co-infection with PRRSV and PCV2b. Significant regions were detected for PCV2b VL, ADG Post Vx, and ADG Post Co-X and results from functional annotation analyses provided biological evidence that supported these statistically associated regions. Results of the functional annotation analyses also supported the many regions with small effects that were detected. Multiple SNPs on SSC7 were significantly associated with PCV2b VL, regardless of prior vaccination for PRRS. However, regions with a significantly different effect on ADG, depending on prior vaccination for PRRS, were detected for ADG Post Vx and Post Co-X. These findings indicate that multiple genomic regions have the potential to be used to select pigs for decreased PCV2b VL following PRRSV/PCV2b co-infection, regardless of prior vaccination for PRRS, but the same is not true for ADG.

This study also introduced a novel approach of using previously-reported QTL to provide evidence for statistically-associated regions from GWAS. Other studies have assessed clustering of health QTL throughout the rice genome [18] and the gene density of QTL regions compared to the rest of the genome in cattle [19], but this is the first study to use a catalog of previously-reported QTL to assess overrepresentation of a QTL category as a means of providing evidence for regions identified from GWAS.

The WUR SNP, a genetic marker for a major QTL for PRRS on SSC4, was identified in a previous study. Results showed that WUR was associated with 15.7% and 11.2% of genetic variation in PRRS VL and weight gain (**WG**) under PRRSV-only infection, respectively [1]. Follow-up studies identified the putative causative mutation in *GBP5* [2], which has been shown to play a role in the innate immune response to infection in mice [20]. In recent years, additional studies have validated the effect of WUR on host response to PRRS using multiple breeds, populations, and following infection with two different PRRSV isolates [3–6]. Results from these studies showed that WUR had a significant effect on PRRS VL following infection with both the NVSL 97-7985 (**NVSL**) and KS2006-72109 (**KS06**) PRRSV isolates, but not WG following infection with KS06 [5]. Authors suggested that this non-significant effect was related to reduced virulence of the KS06 versus the NVSL strain. Taken together, results from these studies showed that WUR had a significant effect on VL with different isolates of PRRSV and for different genetic backgrounds.

A natural follow-up question to these studies was whether WUR also has a significant effect on host response to PRRSV-infection following co-infection with another pathogen. This is a practical question since PRRSV is known to suppress the host immune response, making pigs more susceptible to secondary infections [21]. Co-infection with PRRSV and PCV2b was used as a co-infection model to address this question, given the ubiquitous nature of PCV2b [22, 23], prevalence of PRRSV/PCV2b co-infections in the field (when pigs are not vaccinated for PCV2), and previous experience working with and conducting PCV2b-experimental infection trials. An additional objective was to estimate the effect of WUR on host response to PRRS MLV vaccination since commercial PRRS MLV vaccines are widely used [24, 25]. Although PRRS MLV vaccines are attenuated, modified live virus replicates in pigs post-vaccination.

Results from Dunkelberger et al. [7] showed that WUR had a significant effect on vaccination VL as well as PRRS VL and PCV2b VL of Vx pigs, but not PCV2b VL of Non-Vx pigs. Results also showed that, numerically, the effect of WUR on PRRS VL was greater following primary versus secondary PRRSV exposure, which is consistent with the biological role of *GBP5*. Using these same data, the first objective of the current study was to identify genomic regions other than WUR associated with host response to PRRS MLV vaccination and PRRSV/PCV2b co-infection.

### Genome-wide association studies

#### Viral load

When Vx and Non-Vx pigs were analyzed separately, H3GA0020199, located at SSC7_20, was the only SNP significantly associated with PCV2b VL, and only in Non-Vx pigs. This SNP is located within the gene *KIAA0319*, which has a known role in neuronal growth and migration in humans [26]. This SNP, in addition to several other SNPs located on SSC7 (at 7_19, 7_32, and 7_41) were significantly associated with the main effect of SNP genotype on PCV2b VL. Interestingly, these SNPs are in the vicinity of the swine leukocyte antigens (**SLA**) complex (7_23 to 7_31), one of the most gene-dense regions of the genome which is known to harbor many genes associated with the immune response [27, 28].

A SNP within the SLA was also associated with host response to PCV2b infection in a previous study. This SNP, (referred to as SNP1; name not published) located at 7_28 (SLA Class III) was associated with host response to experimental infection with PCV2b in commercial crossbred pigs [8]. Although the effect of SNP1 did not reach genome-wise significance for the current study, when the effect of SNP1 was fitted as a fixed effect with the effect of other candidates SNPs for PRRS and PCV2b simultaneously, the AA genotype for SNP1 was associated with significantly greater ADG and numerically lower PCV2b VL for Non-Vx pigs [7]. The direction of these effects are consistent with previously reported results, except that Engle et al. [8] did not detect a significant association of SNP1 with ADG post-infection.

There is also evidence of associations of SNPs within the SLA region with host response to PRRSV-infection from other studies, including experimental PRRSV-infection [29] and following a natural PRRSV outbreak [30]. Results from the study conducted by Hess [29] showed that SNPs at 7_26 (SLA Class I) and 7_29 (SLA Class II) were associated with 10-45% of the total genetic variation for PRRS antibody response, depending on infection with the NVSL versus KS06 PRRSV isolate. For the study conducted by Serão et al. [30], SNPs spanning 24 to 30 Mb on SSC7 (harboring SLA Classes I, II, and III) jointly explained 25% of the total genetic variation in antibody response following a natural outbreak in a commercial herd of gestating females.

Despite these sizeable associations of the SLA region with PRRS antibody response, no SNPs within this region, or any other region of the genome, were significantly associated with vaccination VL or PRRS VL for the current study. In general, the lack of significant associations for vaccination VL and PRRS VL is consistent with the conclusion reported by Waide et al. [6] that genomic regions other than WUR explained little to no genetic variation in PRRS VL following experimental infection with the NVSL or KS06 PRRSV isolate. One exception was the 7_30 Mb window, located within the SLA Class II region, which was associated with a small percentage (0.32%) of the total genetic variation in PRRS VL following infection with the KS06 PRRSV isolate [6]. Although not genome-wise significant, a SNP within this same window (ASGA0032151) was the second-most significant SNP *(−log*_*10*_ *P* = 4.25) associated with PRRS VL in Vx pigs for the current study.

#### Average daily gain post vaccination

Contrary to analysis of vaccination VL and PRRS VL, we identified multiple SNPs that were significantly associated with ADG Post Vx and Post Co-X. This finding conflicts with results reported by Waide et al. [6] who reported no significant associations with WG post PRRSV-only infection, other than the WUR region.

Most genomic regions associated with ADG Post Vx in the current study were also associated with ADG in previous studies. However, for all but one of the identified regions, associations with ADG in other studies were in a disease-free environment. For example, the SSC15_129 region associated with ADG of Non-Vx pigs Post Vx, was also associated with ADG in non-challenged pigs by Rückert and Bennewitz [31]. Regions associated with ADG Post Vx of Vx pigs at SSC9_52 (ALGA0052956) and SSC11_53 (WU_10.2_11_53143619 and ALGA0062289) were also associated with ADG in other studies [31, 32]. A region associated with ADG of Non-Vx pigs (WU_10.2_5_5635354) and ADG of Vx pigs Post Vx (WU_10.2_5_5693454), located at 5_5, was the only region associated with ADG Post Vx that was not associated with ADG in a previous study. Therefore, this region may represent a novel QTL for ADG. Interestingly, this SNP (WU_10.2_5_5693454) is located within the growth factor receptor-bound protein 2 (**GRB2**)-reltated adaptor protein 2 gene in pigs, also known as GRB2-related adaptor downstream of Shc **(GADS)**. In humans, GADS is involved in the formation of the T cell receptor complex [33] and has a known role in T cell development and signaling [34]. However, there was no evidence that this SNP was significantly associated with any of the VL traits.

None of the SNPs that were significantly associated with ADG of Non-Vx pigs were also significantly associated with ADG of Vx pigs. However, several other SNPs had a significant effect on ADG Post Vx, regardless of vaccination status, which agrees with the high, positive (0.92 ± 0.92) genetic correlation identified between ADG of Vx and Non-Vx pigs Post Vx reported in our previous study using these same data [7]. These SNPs, with significant main effects, were located at 6_108, 7_10, 7_81, 7_82, and 18_4, all of which were associated with ADG in previous studies [35–38]. Given the significant main effect detected for these SNPs, these five SNPs may be used to select pigs for improved host response to PRRSV/PCV2b co-infection, regardless of prior vaccination for PRRS. Interestingly, the SNP located on SSC6 (ALGA0036437) is located within the Ring Finger Protein 125 **(** ***RNF125)*** gene, which is a negative regulator of the RIG-1 like receptor signaling pathway [39]. The RIG-1 like receptor is a known recognition receptor of RNA viruses. Activated RIG-1 like protein signals for the production of cytokines, including type I IFN in the innate immune response pathway [39]. Suggestive evidence of a significant *(−log*_*10*_ *P* = 3.16) association of this SNP with the main effect of SNP genotype on PRRS VL was also detected.

The effect of ASGA0077518 (17_57) on ADG Post Vx depended on prior vaccination for PRRS, where the effect of this SNP on ADG was near genome-wise significance for Vx pigs, but not Non-Vx pigs. The nearest QTL for ADG reported in a previous study spans 17_64 to 17_66 [40]. During the vaccination period, ADG of Vx pigs represented growth under PRRSV-infection (albeit a modified PRRSV infection), while ADG of Non-Vx pigs represented growth under non-challenged conditions. Therefore, the significant interaction identified for ASGA0077518 indicates that this SNP had a significantly different effect on growth rate Post Vx, depending on PRRS vaccination status.

#### Average daily gain post co-infection

Similar to analysis of ADG Post Vx, several SNPs were also significantly associated with ADG Post Co-X, including the only SNP that was previously associated with growth under disease challenged conditions in a separate study [3]. This SNP, H3GA0020408, is located in the SLA region (7_27) and was associated with ADG of Non-Vx pigs Post Co-X. This same 1-Mb window was also associated with WG following PRRSV-only infection for analysis of PRRS Host Genetics Consortium trials 1-8 [3].

Other significant SNPs associated with ADG Post Co-X included a SNP (WU_10.2_15_140171163) located at 15_140. This same region was associated with ADG of non-challenged pigs in a previous study [41]. MARC0021766, located at 1_47, was associated with ADG of Vx pigs and this same region was also associated with ADG in a previous study [31]. Associations of SNPs located on SSC14 (H3GA0040428 and ALGA0077929) at 61 and 62 Mb, respectively, with ADG of Vx pigs have not been previously reported and may represent novel QTL for ADG under challenged conditions. No candidate genes were identified for H3GA0040428, but ALGA0077929 is located within the Pecanex Homolog 2 (***PCNXL2*** *)* gene. In humans, *PCNXL2* has been associated with susceptibility to colorectal cancer [42].

Similar to results for ADG Post Vx, none of the SNPs that were significantly associated with ADG of Non-Vx pigs were also significantly associated with ADG of Vx pigs Post Co-X. However, several other SNPs had a significant effect on ADG regardless of prior vaccination for PRRS, which agrees with our previous finding of a moderate to high (0.75 ± 0.37) genetic correlation between ADG of Vx and Non-Vx pigs [7]. These SNPs included MARC0021766 on SSC1 (significant for Vx pigs) and WU_10.2_15_140171163 on SSC15 (significant for Non-Vx pigs). Therefore, MARC0021766 and WU_10.2_15_140171163 can be used to select pigs for improved host response to PRRSV/PCV2b co-infection, regardless of prior vaccination for PRRS. WU_10.2_4_6084304, located at 4_6, was also associated with ADG in a previous study [38].

### Protein pathway analyses

In addition to identifying genomic regions associated with host response to PRRS vaccination and PRRSV and PCV2b co-infection, another objective of this study was to provide biological evidence for regions identified from GWAS. PANTHER software was used to test for enrichment of genes near SNPs associated with each trait.

#### Viral load

Pathways enriched in genes near SNPs associated with vaccination VL included cell proliferation, cell movement, cell signaling, and cytokine signaling. Enrichment of these pathways is consistent with the literature regarding PRRS MLV vaccination response. Compared to infection with a field isolate of PRRSV, PRRS MLV vaccination is known to result in a delayed humoral and cell-mediated immune response [43]. Cell-mediated immunity is often characterized by lymphocyte proliferation and increased cytokine production, mainly production of IFN-γ [44, 45]. The pathways “cell proliferation” and “cell signaling” reflect these processes.

Consistent with results reported by Waide et al. [6], the G-protein coupled receptor signaling pathway was enriched for genes near SNPs associated with PRRS VL of Vx pigs. Enrichment of this pathway is an interesting result since this protein receptor class plays a role in T cell immunity, including T cell migration by regulating chemotaxis [46]. Results from a previous study also showed that AA and AB pigs differ regarding utility of the G-protein coupled receptor pathway. Pigs with the AA genotype showed extended expression of this pathway (which is essential for the phosphoinositide 3 kinase pathway which is related to PRRS virus entry) compared to AB pigs, thereby providing greater opportunity for PRRSV entry into host cells [47]. No pathways were enriched in genes near SNPs associated with the main effect of SNP genotype for PRRS VL, which is consistent with GWAS results for this trait.

Immune-related pathways were also enriched for genes near SNPs associated with PCV2b VL, including but not limited to: cellular defense response, cell-to-cell adhesion, and nucleobase metabolic processes for Vx pigs only, and the chromatin organization pathway for both Vx and Non-Vx pigs. This result agrees with the finding that several SNPs near the SLA region were significant for the main effects of SNP on PCV2b VL. It is also consistent with the high, positive genetic correlation between PCV2b VL of Vx and Non-Vx pigs (0.99 ± 0.94) reported for our previous analyses of these same data [7]. Based on this estimate, it is expected that the same genomic regions, and therefore the same protein pathways, are associated with PCV2b VL Post Co-X, regardless of previous vaccination for PRRS.

For PCV2b VL, genes near SNPs associated with the main effect of SNP were enriched for primary metabolic process and response to IFN-γ. The “primary metabolic process” pathway was previously found to be associated with PRRS VL following infection with the KS06 and NVSL PRRSV isolates [6]. This pathway likely reflects the need for metabolizable energy to mount and maintain an immune response [48]. Enrichment of genes in the “response to IFN-γ” pathway is particularly interesting, given the well-known role of IFN-γ in response to viral infection. There is evidence that IFN-γ production increases with increasing replication of PCV2b [49] and that PCV2b replication increases in pigs that are already infected with PRRSV [50, 51].

#### Average daily gain

Results of protein pathways analyses showed enrichment of metabolism-related pathways for genes near SNPs associated with ADG of Vx and Non-Vx pigs Post Co-X. Lipid transport was also enriched for genes near SNPs associated with the interaction effect of SNP genotype by VxStatus on ADG Post Co-X. Metabolic processes are clearly associated with growth rate and such pathways were also enriched for WG following PRRSV-only infection in a previous study [6].

For the protein pathway analyses, it is important to note that all analyses were performed using the 10.2 build of the swine genome. Therefore, repeating these analyses using the newer (11.1) build could change results for any regions previously misassembled and/or regions for which additional annotation information is now available.

### QTL and SNP tests

The QTL and SNP Tests proposed in this study were then used to provide another piece of evidence for regions identified from GWAS. A significant result for the QTL Test indicated that SNPs identified from GWAS mapped to significantly more unique trait QTL (i.e. health or growth QTL) reported in previous studies than expected by chance. The SNP Test was designed to answer a similar question, where the objective was to assess whether the proportion of SNPs within a SNP list that mapped to health or growth QTL was greater than expected by chance. In general, similar results were obtained for the QTL and SNP Tests for analyses of each trait.

#### Viral load

Results of the QTL Test for the main and interaction effects of SNP for PRRS and PCV2b VL were consistent with results of the SNP Test, except that for T3, the main effect of SNP was not significant for SNPs associated with PRRS VL for the SNP Test. When PRRS and PCV2b VL were analyzed separately by vaccination status, every SNP list showed significant overrepresentation of health QTL, except for T3 for PRRS VL of Non-Vx pigs. These results were consistent with those obtained for the SNP Test, except that none of the SNP lists were significant for analysis of PRRS VL of Non-Vx pigs. This was driven by the fact that, compared to PRRS VL of Vx pigs, similar numbers of SNPs were identified for each SNP list, but fewer SNPs mapped to health QTL for Non-Vx versus Vx pigs.

Collectively, results of these tests indicate that regions associated with PRRSV and PCV2b VL showed significant overrepresentation for health QTL identified from previous studies, especially when Vx and Non-Vx pigs were analyzed separately. Results of the SNP Test show that mapping of SNPs to health QTL was non-random. Non-significant results obtained for the main/interaction effects of SNP likely reflect noise since relaxed –log_10_ *p*-value thresholds were used to construct the SNP lists, thereby including SNPs with smaller effects on the trait of interest. Non-significant results for the main/interaction effects of SNP on PRRS VL and for the interaction effect for PCV2b VL indeed appear to be consistent with GWAS results for these traits, for which no SNPs reached genome-wise significance for these effects.

#### Average daily gain

Few significant results were obtained for the QTL Test for ADG. Only two SNP lists, both for the main effect of SNP on ADG Post Vx, showed a significant result. Overrepresentation of growth QTL was not detected for any of the SNP lists for SNPs associated with ADG Post Co-X, except for T3 for ADG of Non-Vx pigs. An additional SNP list (T2) showed significant overrepresentation of growth QTL for ADG of Non-Vx pigs Post Vx for the SNP Test. Possibly, significant results for the QTL and SNP Tests were obtained for Non-Vx pigs Post Vx only because this trait represents growth in a healthy environment, which is the same condition under which growth traits were measured for the majority of previously-identified growth QTL. Therefore, non-significant results for analysis of ADG of Vx pigs Post Vx or ADG Post Co-X might indicate the identification of novel QTL for ADG, and/or QTL specific to growth under challenge.

Collectively, results of the QTL and SNP Tests for SNPs associated with ADG indicate significant overrepresentation of growth QTL for growth rate under non-challenged conditions, but not under challenged conditions. For growth under challenged conditions, significant results for the SNP Test also indicate non-random associations of SNPs mapped to growth QTL.

The QTL Test and SNP Test used in this study are novel approaches used to provide evidence for statistically associated regions from GWAS. Although these tests have shown to provide valuable evidence for regions identified in this study, it is also important to note that results obtained from these tests are contingent on several factors. For example, several necessary steps for preparing the filtered QTL list are subject to modification, including the criteria used to determine a “unique” QTL entry and the length of the QTL interval used to retain unique entries. In addition, a limiting factor of the QTL and SNP Tests is that it is only possible to test for significant overrepresentation of QTL using QTL that have already been identified. Therefore, a non-significant result merely implies that QTL of a particular category are not significantly overrepresented for a subset of SNPs based on the current catalog of QTL for the pig genome. Consequently, non-significant results are obtained for SNPs mapping to undiscovered QTL.

Other possible limitations are that results are also subject to the window size used to identify genes mapping to significant SNPs, as well as the significance thresholds used to construct the SNP lists. For the current study, 1-Mb SNP windows were used to allow for the possibility of trans-acting QTL and SNP list thresholds were selected to be consistent with the procedure described by Waide et al. [6], as previously mentioned. These thresholds were purposefully selected to include SNPs that did not reach genome-wise significance in order to capture the effects of all SNPs affecting the trait of interest, including those with small effects. This was important because disease-related traits are considered complex traits, and are therefore assumed to be controlled by many loci with small effects [52].

## Conclusions

In conclusion, other than WUR, several other genomic regions were associated with host response to PRRS MLV vaccination and co-infection with PRRSV and PCV2b, but in general, host response was highly polygenic. Multiple SNPs near the SLA region were associated with PCV2b VL, regardless of previous vaccination for PRRS. Several regions associated with ADG Post Vx and Post Co-X were also identified, but SNPs with a significantly different effect on ADG, depending on vaccination status, were identified for ADG during both periods. Taken together, results indicate that multiple SNPs near the SLA region have the potential to be used to select pigs for decreased PCV2b VL following PRRSV/PCV2b co-infection, but that different SNPs were associated with ADG following PRRS vaccination and PRRSV/PCV2b co-infection, depending on previous vaccination for PRRS.

Results from the protein pathway enrichment analyses supported GWAS results, showing that immune-related pathways were enriched for genes near SNPs associated with vaccination VL, PRRS VL, and PCV2b VL and that metabolic pathways were enriched for genes near SNPs associated with ADG. Results of the QTL and SNP Tests provided additional evidence for the identified regions and similar results were obtained for both tests. Results showed that SNPs associated with vaccination VL, PRRS VL, and PCV2b VL were significantly overrepresented for health QTL when Vx and Non-Vx pigs were analyzed separately and results of the SNP Test showed that mapping of SNPs to health QTL was non-random. Results for ADG showed that, for Non-Vx pigs prior to co-infection, SNPs associated with ADG were significantly overrepresented for growth QTL and results of the SNP Test showed that mapping of SNPs to growth QTL was non-random. These findings likely reflect the fact that most QTL used for these tests were associated with growth under non-challenged conditions.

Collectively, results of functional annotation analyses provide valuable insight regarding the biological pathways underlying host response to PRRS MLV vaccination and PRRSV/PCV2b co-infection, biological evidence for regions statistically associated with the traits of interest, and a means of summarizing GWAS results for complex traits, including host response to disease challenge.

## Additional file

Additional file 1: Table S1. Number of independent principal components per chromosome required to capture 99.5% of the genetic variation. **Table S2.** Number of GO terms corresponding to lists of SNPs associated with each trait. **Table S3.** Number of GO terms corresponding to SNP lists associated with the main/interaction effect of SNP. **Table S4.** Significantly enriched GO terms for genes near SNPs associated with ADG following PRRS vaccination. **Table S5.** Significantly enriched GO terms for genes near SNPs associated with PRRS VL following PRRSV/PCV2b co-infection. **Table S6.** Significantly enriched GO terms for genes near SNPs associated with PCV2b VL following PRRSV/PCV2b co-infection. **Table S7.** Significantly enriched GO terms for genes near SNPs associated with ADG following PRRSV/PCV2b co-infection. **Table S8.** QTL Test results for SNPs associated with PRRS and PCV2b VL. **Table S9.** QTL Test results for SNPs associated with ADG following PRRS vaccination and PRRSV/PCV2b co-infection. **Table S10.** SNP Test results for SNPs associated with PRRS and PCV2b VL. **Table S11.** SNP Test results for SNPs associated with ADG following PRRS vaccination and PRRSV/PCV2b co-infection. (DOCX 74 kb)

## Abbreviations

ADG: Average daily gain
Dpi: Days post-infection
GADS: GRB2-related adaptor downstream of Shc
*GBP5*: Guanylate binding protein 5
GO: Gene ontology
GRB2: growth factor receptor-bound protein 2
GRM: Genomic relationship matrix
IFN: Interferon
Interaction effect: The interaction of SNP genotype with vaccination status
KS06: KS2006-72109
Main effect: The effect of each SNP averaged across vaccination status
Mb: Megabase
Non-Vx: Non-vaccinated
NVSL: NVSL 97-7985.
*PCNXL2*: Pecanex Homolog 2
PCV2b: Porcine circovirus type 2b
Post Co-X: Post co-infection
Post Vx: Post vaccination
PRRS: Porcine reproductive and respiratory syndrome
PRRSV: PRRS virus
QTL: Quantitative trait locus
*RNF125*: Ring Finger Protein 125
SLA: Swine leukocyte antigens
SNP: Single nucleotide polymorphism
SSC: *Sus scrofa* chromosome
T2: Lists of SNPs with a –log_10_ *p*-value ≥2
T2.5: Lists of SNPs with a –log_10_ p-value ≥2.5
T3: Lists of SNPs with a –log_10_ p-value ≥3
VL: Viral load
Vx: Vaccinated
WG: Weight gain
WUR: WUR1000125
