# Supplementary material for: Genomic regions associated with host response to porcine reproductive and respiratory syndrome vaccination and co-infection in nursery pigs
Source: BMC Genomics. 2017 Nov 13;18:865. doi: 10.1186/s12864-017-4182-8 (PMC5682865; doi:10.1186/s12864-017-4182-8)
Supplement: Additional file 1: Table S1. — Number of independent principal components per chromosome required to capture 99.5% of the genetic variation. Table S2. Number of GO terms corresponding to lists of SNPs associated with each trait. Table S3. Number of GO terms corresponding to SNP lists associated with the main/interaction effect of SNP. Table S4. Significantly enriched GO terms for genes near SNPs associated with ADG following PRRS vaccination. Table S5. Significantly enriched GO terms for genes near SNPs associated with PRRS VL following PRRSV/PCV2b co-infection. Table S6. Significantly enriched GO terms for genes near SNPs associated with PCV2b VL following PRRSV/PCV2b co-infection. Table S7. Significantly enriched GO terms for genes near SNPs associated with ADG following PRRSV/PCV2b co-infection. Table S8. QTL Test results for SNPs associated with PRRS and PCV2b VL. Table S9. QTL Test results for SNPs associated with ADG following PRRS vaccination and PRRSV/PCV2b co-infection. Table S10. SNP Test results for SNPs associated with PRRS and PCV2b VL. Table S11. SNP Test results for SNPs associated with ADG following PRRS vaccination and PRRSV/PCV2b co-infection. (DOCX 74 kb) [file 12864_2017_4182_MOESM1_ESM.docx]

**Table S1:** **Number of independent principal components per chromosome required to capture 99.5% of the genetic variation.**

| **Chromosome** | **# SNPs^1^** | **# Principal Components** |
| --- | --- | --- |
| **1** | 5,730 | 2,466 |
| **2** | 3,784 | 1,677 |
| **3** | 3,398 | 1,569 |
| **4** | 3,623 | 1,574 |
| **5** | 2,792 | 1,263 |
| **6** | 4,330 | 1,862 |
| **7** | 3,680 | 1,570 |
| **8** | 3,309 | 1,503 |
| **9** | 3,576 | 1,634 |
| **10** | 2,564 | 1,180 |
| **11** | 2,176 | 994 |
| **12** | 2,309 | 970 |
| **13** | 4,241 | 1,740 |
| **14** | 3,866 | 1,504 |
| **15** | 3,360 | 1,480 |
| **16** | 2,210 | 975 |
| **17** | 1,978 | 855 |
| **18** | 1,630 | 672 |
| **X** | 2,581 | 783 |
| **Y** | 10 | 1 |

^1^SNPs, single nucleotide polymorphisms.

**Table S2:** **Number of GO terms corresponding to lists of SNPs associated with each trait.**

| **Infection Period^1^** | **Trait** | **Vaccination Status** | **SNP List^2^** | **# of SNPs above threshold** | **Total # Ensembl IDs** | **# Ensembl IDs with GO annotation** |
| --- | --- | --- | --- | --- | --- | --- |
|  | **Vaccination VL^3^** | **Vx** | 2 | 392 | 1,101 | 919 |
| **Post Vaccination** |  |  | 2.5 | 124 | 472 | 403 |
|  |  |  | 3 | 51 | 147 | 128 |
|  | **ADG** | **Non-Vx** | 2 | 828 | 1,649 | 1,368 |
|  |  |  | 2.5 | 355 | 707 | 562 |
|  |  |  | 3 | 161 | 297 | 232 |
|  |  | **Vx** | 2 | 769 | 2,017 | 1,664 |
|  |  |  | 2.5 | 295 | 863 | 717 |
|  |  |  | 3 | 120 | 376 | 314 |
| **Post**  **Co-Infection** | **PRRS VL** | **Non-Vx** | 2 | 534 | 1,309 | 1,103 |
|  |  |  | 2.5 | 164 | 386 | 330 |
|  |  |  | 3 | 36 | 99 | 84 |
|  |  | **Vx** | 2 | 582 | 1,467 | 1,197 |
|  |  |  | 2.5 | 170 | 614 | 502 |
|  |  |  | 3 | 50 | 231 | 181 |
|  | **PCV2b VL** |  | 2 | 723 | 1,674 | 1,376 |
|  |  | **Non-Vx** | 2.5 | 232 | 605 | 496 |
|  |  |  | 3 | 66 | 229 | 177 |
|  |  |  | 2 | 566 | 1,341 | 1,106 |
|  |  | **Vx** | 2.5 | 178 | 483 | 393 |
|  |  |  | 3 | 51 | 161 | 130 |
|  | **ADG** |  | 2 | 577 | 1,450 | 1,190 |
|  |  | **Non-Vx** | 2.5 | 227 | 667 | 534 |
|  |  |  | 3 | 119 | 397 | 316 |
|  |  |  | 2 | 978 | 2,314 | 1,878 |
|  |  | **Vx** | 2.5 | 502 | 1,260 | 1,025 |
|  |  |  | 3 | 285 | 736 | 602 |

^1^Infection Period: Pigs were vaccinated (**Vx**) or not (**Non-Vx**) for PRRS prior to co-infection of PRRSV with PCV2b 28 days later.

^2^SNP List: List of SNPs from the GWAS with a –log_10_ p-value above 2, 2.5, or 3.

^3^VL: Calculated as the area under the curve of serum viremia between -28 and 0, 0 and 21, or 0 and 42 dpi for vaccination VL, PRRS VL, and PCV2b VL, respectively.

**Table S3: Number of GO terms corresponding to SNP lists associated with the main/interaction effect of SNP.**

| **Infection Period^1^** | **Trait** | **Effect^2^** | **SNP List^3^** | **# SNPs above threshold** | **Total # Ensembl IDs** | **# Ensembl IDs with GO annotation** |
| --- | --- | --- | --- | --- | --- | --- |
|  |  | **Main** | 2 | 902 | 1,920 | 1,590 |
| **Post Vaccination** | **ADG** |  | 2.5 | 365 | 859 | 694 |
|  |  |  | 3 | 141 | 449 | 371 |
|  |  |  | 2 | 687 | 1,556 | 1,244 |
|  |  | **Interaction** | 2.5 | 251 | 562 | 444 |
|  |  |  | 3 | 110 | 284 | 231 |
| **Post**  **Co-Infection** | **PRRS VL^4^** | **Main** | 2 | 528 | 1,320 | 1,078 |
|  |  |  | 2.5 | 168 | 479 | 380 |
|  |  |  | 3 | 37 | 136 | 116 |
|  |  |  | 2 | 516 | 1,313 | 1,066 |
|  |  | **Interaction** | 2.5 | 159 | 487 | 398 |
|  |  |  | 3 | 42 | 125 | 108 |
|  | **PCV2b VL** | **Main** | 2 | 823 | 1,644 | 1,344 |
|  |  |  | 2.5 | 350 | 743 | 596 |
|  |  |  | 3 | 160 | 365 | 290 |
|  |  |  | 2 | 520 | 1,150 | 918 |
|  |  | **Interaction** | 2.5 | 156 | 363 | 291 |
|  |  |  | 3 | 44 | 98 | 69 |
|  | **ADG** | **Main** | 2 | 870 | 2,084 | 1,687 |
|  |  |  | 2.5 | 364 | 1,093 | 886 |
|  |  |  | 3 | 176 | 555 | 449 |
|  |  | **Interaction** | 2 | 682 | 1,592 | 1,306 |
|  |  |  | 2.5 | 291 | 727 | 602 |
|  |  |  | 3 | 120 | 357 | 300 |

^1^Infection Period: Pigs were vaccinated (**Vx**) or not (**Non-Vx**) for PRRS prior to co-infection of PRRSV with PCV2b 28 days later.

^2^Effect: The effect of SNP across groups vaccinated, or not, for PRRS (**main**) versus the effect of SNP interacting with vaccination status (**interaction**).

^3^SNP List: List of SNPs from the GWAS with a –log_10_ p-value above 2, 2.5, or 3.

^4^VL: Calculated as the area under the curve of serum viremia between 0 and 21 or 0 and 42 dpi for PRRS VL and PCV2b VL, respectively.

**Table S4:** **Significantly enriched GO terms for genes near SNPs associated with ADG following PRRS vaccination**.

| **Effect^1^** | **SNP List^2^** | **GO term** | **Fold change** | **P-value^3^** |
| --- | --- | --- | --- | --- |
| **Main** | **2** | Sensory perception of smell | 0.47 | 2.2E-4 |
|  | **2.5** | Sensory perception of smell | < 0.20 | 9.0E-6 |
|  | **3** | Sensory perception of smell | < 0.20 | 5.0E-4 |
| **Interaction** | **2** | Response to stimulus | 0.76 | 3.7E-2 |
|  |  | Regulation of biological process | 0.58 | 1.3E-6 |
|  |  | Biological regulation | 0.64 | 1.3E-5 |
|  |  | G-protein coupled receptor signaling pathway | 0.51 | 4.2E-3 |
|  |  | Sensory perception of smell | < 0.20 | 9.6E-17 |
|  |  | Sensory perception of chemical stimulus | 0.28 | 5.5E-10 |
|  |  | Sensory perception | 0.52 | 1.8E-4 |
|  |  | Neurological system process | 0.64 | 3.2E-3 |
|  |  | System process | 0.68 | 1.3E-2 |
|  |  | Single-multicellular organism process | 0.73 | 3.6E-2 |
|  |  | Multicellular organismal process | 0.73 | 4.7E-2 |
|  | **2.5** | G-protein coupled receptor signaling pathway | 0.24 | 6.6E-3 |
|  |  | Cell surface receptor signaling pathway | 0.46 | 4.6E-2 |
|  |  | Sensory perception of smell | < 0.20 | 3.9E-5 |
|  |  | Sensory perception of chemical stimulus | < 0.20 | 4.2E-6 |
|  |  | Sensory perception | 0.32 | 4.0E-3 |
|  | **3** | Secondary metabolic process | < 0.20 | 8.7E-3 |
|  |  | Endocytosis | < 0.20 | 3.3E-2 |
|  |  | Sensory perception of chemical stimulus | < 0.20 | 1.6E-2 |

^1^Effect: The effect of SNP across groups vaccinated, or not, for PRRS (**main**) versus the effect of SNP interacting with vaccination status (**interaction**).

^2^SNP List: List of SNPs from the GWAS with a –log_10_ p-value above 2, 2.5, or 3.

^3^P-value: Bonferroni corrected p-value.

**Table S5:** **Significantly enriched GO terms for genes near SNPs associated with PRRS VL following PRRSV/PCV2b co-infection.**

| **Effect^1^** | **SNP List^2^** | **GO term** | **Fold change** | **P-value^3^** |
| --- | --- | --- | --- | --- |
| **Main** | 2 | -- | -- | -- |
|  | 2.5 | -- | -- | -- |
|  | 3 | -- | -- | -- |
| **Interaction** | 2 | Response to stimulus | 1.29 | 2.1E-2 |
|  |  | Cellular protein modification process | 0.44 | 8.2E-4 |
|  | 2.5 | Chromatin organization | 3.11 | 1.9E-2 |
|  | 3 | -- | -- | -- |

^1^Effect: The effect of SNP across groups vaccinated, or not, for PRRS (**main**) versus the effect of SNP interacting with vaccination status (**interaction**).

^2^SNP List: List of SNPs from the GWAS with a –log_10_ p-value above 2, 2.5, or 3.

^3^P-value: Bonferroni corrected p-value.

**Table S6:** **Significantly enriched GO terms for genes near SNPs associated with PCV2b VL following PRRSV/PCV2b co-infection**

| **Effect^1^** | **SNP List^2^** | **GO term** | **Fold change** | **P-value^3^** |
| --- | --- | --- | --- | --- |
| **Main** | 2 | -- | -- | -- |
|  | 2.5 | Response to interferon-gamma | 5.27 | 1.6E-2 |
|  |  | Chromatin organization | 2.77 | 9.0E-3 |
|  | 3 | Chromatin organization | 4.23 | 4.4E-4 |
|  |  | Primary metabolic process | 1.36 | 4.9E-2 |
| **Interaction** | 2 | Regulation of biological process | 0.55 | 2.1E-5 |
|  |  | Biological regulation | 0.65 | 1.7E-3 |
|  |  | Sensory perception of chemical stimulus | < 0.20 | 4.0E-15 |
|  |  | Sensory perception | 0.21 | 1.8E-11 |
|  |  | Neurological system process | 0.58 | 3.6E-3 |
|  |  | System process | 0.64 | 2.2E-2 |
|  |  | Single-multicellular organism process | 0.64 | 3.4E-3 |
|  |  | Multicellular organismal process | 0.64 | 3.1E-3 |
|  | 2.5 | Transport | 1.73 | 4.6E-2 |
|  |  | Regulation of biological process | 0.41 | 1.1E-2 |
|  |  | G-protein coupled receptor signaling pathway | < 0.20 | 2.4E-2 |
|  | 3 | -- | -- | -- |

^1^Effect: The effect of SNP across groups vaccinated, or not, for PRRS (**main**) versus the effect of SNP interacting with vaccination status (**interaction**).

^2^SNP List: List of SNPs from the GWAS with a –log_10_ p-value above 2, 2.5, or 3.

^3^P-value: Bonferroni corrected p-value.

**Table S7:** **Significantly enriched GO terms for genes near SNPs associated with ADG following PRRSV/PCV2b co-infection**.

| **Effect^1^** | **SNP List^2^** | **GO term** | **Fold change** | **P-value^3^** |
| --- | --- | --- | --- | --- |
| **Main** | 2 | -- | -- | -- |
|  | 2.5 | Chromatin organization | 2.59 | 1.3E-3 |
|  | 3 | Chromatin organization | 4.20 | 1.6E-6 |
| **Interaction** | 2 | Localization | 1.31 | 3.4E-2 |
|  |  | Cell surface receptor signaling pathway | 0.65 | 1.2E-2 |
|  |  | Sensory perception of smell | 0.20 | 1.3E-10 |
|  |  | Sensory perception of chemical stimulus | 0.31 | 2.1E-9 |
|  |  | Sensory perception | 0.41 | 5.9E-8 |
|  |  | Neurological system process | 0.68 | 2.3E-2 |
|  |  | System process | 0.70 | 1.8E-2 |
|  |  | Single-multicellular organism process | 0.68 | 1.2E-3 |
|  |  | Multicellular organismal process | 0.68 | 1.1E-3 |
|  | 2.5 | Lipid transport | 4.06 | 1.4E-2 |
|  |  | Chromatin organization | 2.62 | 2.8E-2 |
|  |  | Sensory perception of chemical stimulus | 0.38 | 1.0E-2 |
|  | 3 | -- | -- | -- |

^1^Effect: The effect of SNP across groups vaccinated, or not, for PRRS (**main**) versus the effect of SNP interacting with vaccination status (**interaction**).

^2^SNP List: List of SNPs from the GWAS with a –log_10_ p-value above 2, 2.5, or 3.

^3^P-value: Bonferroni corrected p-value.

**Table S8:** **QTL Test results for SNPs associated with PRRS and PCV2b VL.**

| **Trait^1^** | **SNP List^2^** | **# of SNPs above threshold** | **# of health QTL in region^3^** | **Total # QTL in region^4^** | **P-value** |
| --- | --- | --- | --- | --- | --- |
| **PRRS VL** **Non-Vx** | 2 | 534 | 295 | 1039 | **3.3E-18** |
|  | 2.5 | 164 | 98 | 315 | **2.9E-9** |
|  | 3 | 36 | 14 | 66 | 0.26 |
| **PRRS VL Vx** | 2 | 582 | 353 | 1432 | **6.7E-12** |
|  | 2.5 | 170 | 122 | 473 | **4.2E-6** |
|  | 3 | 50 | 78 | 262 | **7.9E-7** |
| **PCV2b VL Non-Vx** | 2 | 723 | 432 | 1887 | **1.7E-9** |
|  | 2.5 | 232 | 207 | 856 | **5.2E-7** |
|  | 3 | 66 | 77 | 304 | **4.0E-4** |
| **PCV2b VL Vx** | 2 | 566 | 349 | 1694 | **6.0E-4** |
|  | 2.5 | 178 | 174 | 789 | **6.0E-4** |
|  | 3 | 51 | 67 | 243 | **6.7E-5** |

^1^Trait: Each trait was recorded on pigs vaccinated (**Vx**) or not (**Non-Vx**) for PRRS prior to co-infection of PRRSV with PCV2b 28 days later.

^2^SNP List: Lists of SNPs from the GWAS with a –log_10_ p-value above 2, 2.5 or 3.

^3^The total number of health QTL in the genome (after filtering) is 1,732.

^4^The total number of QTL in the genome (i.e. across all QTL types after filtering) is 9,892.

**Table S9:** **QTL Test results for SNPs associated with ADG following PRRS vaccination and PRRSV/PCV2b co-infection**.

| **Infection Period** | **Trait^1^** | **SNP List^2^** | **# of SNPs above threshold** | **# of growth QTL in region^3^** | **Total # of QTL in region^4^** | **P-value** |
| --- | --- | --- | --- | --- | --- | --- |
|  | **Non-Vx** | 2 | 828 | 71 | 1456 | 0.56 |
| **Post Vaccination** |  | 2.5 | 355 | 33 | 603 | 0.30 |
|  |  | 3 | 161 | 20 | 267 | **0.04** |
|  | **Vx** | 2 | 769 | 84 | 1514 | 0.15 |
|  |  | 2.5 | 295 | 35 | 614 | 0.21 |
|  |  | 3 | 120 | 13 | 220 | 0.29 |
|  | **Non-Vx** | 2 | 577 | 62 | 1352 | 0.74 |
| **Post**  **Co-Infection** |  | 2.5 | 227 | 35 | 785 | 0.75 |
|  |  | 3 | 119 | 19 | 489 | 0.88 |
|  | **Vx** | 2 | 978 | 110 | 2156 | 0.37 |
|  |  | 2.5 | 502 | 62 | 1196 | 0.36 |
|  |  | 3 | 285 | 40 | 781 | 0.43 |

^1^Trait: Each trait was recorded on pigs vaccinated (**Vx**) or not (**Non-Vx**) for PRRS prior to co-infection of PRRSV with PCV2b 28 days later.

^2^SNP List: Lists of SNPs from the GWAS with a –log_10_ p-value above 2, 2.5 or 3.

^3^The total number of growth QTL in the genome (after filtering) is 488.

^4^The total number of QTL in the genome (i.e. across all QTL types after filtering) is 9,892.

**Table S10: SNP Test results for SNPs associated with PRRS and PCV2b VL.**

| **Trait^1^** | **SNP List^2^** | **# SNPs mapping to health QTL in SNP list^3^** | **# SNPs**  **in SNP list^4^** | **P-value** |
| --- | --- | --- | --- | --- |
| **PRRS VL** **Non-Vx** | 2 | 125 | 534 | 0.38 |
|  | 2.5 | 40 | 164 | 0.34 |
|  | 3 | 7 | 36 | 0.74 |
| **PRRS VL Vx** | 2 | 205 | 582 | **7.0E-12** |
|  | 2.5 | 53 | 170 | **7.0E-3** |
|  | 3 | 22 | 50 | **7.0E-4** |
| **PCV2b VL Non-Vx** | 2 | 208 | 723 | **1.1E-4** |
|  | 2.5 | 67 | 232 | **0.02** |
|  | 3 | 25 | 66 | **4.0E-3** |
| **PCV2b VL Vx** | 2 | 153 | 566 | **0.01** |
|  | 2.5 | 51 | 178 | **0.04** |
|  | 3 | 14 | 51 | 0.26 |

^1^Trait: Each trait was recorded on pigs vaccinated (**Vx**) or not (**Non-Vx**) for PRRS prior to co-infection of PRRSV with PCV2b 28 days later.

^2^SNP List: Lists of SNPs from the GWAS with a –log_10_ p-value above 2, 2.5 or 3.

^3^The number of unique SNPs mapping to health QTL in the genome is 14,063.

^4^The total number of SNPs used for the GWAS was 61,729.

**Table S11:** **SNP Test results for SNPs associated with ADG following PRRS vaccination and PRRSV/PCV2b co-infection**.

| **Infection Period** | **Trait^1^** | **SNP List^2^** | **# SNPs mapping to growth QTL in SNP list^3^** | **# SNPs**  **in SNP list^4^** | **P-value** |
| --- | --- | --- | --- | --- | --- |
| **Post Vaccination** | **Non-Vx** | 2 | 163 | 828 | **2.0E-4** |
|  |  | 2.5 | 60 | 355 | 0.18 |
|  |  | 3 | 46 | 161 | **8.7E-6** |
|  | **Vx** | 2 | 115 | 769 | 0.55 |
|  |  | 2.5 | 43 | 295 | 0.62 |
|  |  | 3 | 19 | 120 | 0.44 |
| **Post Co-Infection** | **Non-Vx** | 2 | 72 | 577 | 0.97 |
|  |  | 2.5 | 31 | 227 | 0.75 |
|  |  | 3 | 18 | 119 | 0.53 |
|  | **Vx** | 2 | 154 | 978 | 0.29 |
|  |  | 2.5 | 65 | 502 | 0.92 |
|  |  | 3 | 40 | 285 | 0.71 |

^1^Trait: Each trait was recorded on pigs vaccinated (**Vx**) or not (**Non-Vx**) for PRRS prior to co-infection of PRRSV with PCV2b 28 days later.

^2^SNP List: Lists of SNPs from the GWAS with a –log_10_ p-value above 2, 2.5 or 3.

^3^The number of unique SNPs mapping to growth QTL in the genome is 9,294.

^4^The total number of SNPs used for the GWAS was 61,729.
